# Effects of the serjania erecta and zeyheria montana ethanol extracts in experimental pulpitis in rats: A histological study

**DOI:** 10.4317/medoral.18470

**Published:** 2012-12-10

**Authors:** Patrícia M. Nossa, Leandro C. Guenka, Lucélio B. Couto, Danyel E. da-Cruz-Perez

**Affiliations:** 1 DDS, MSc, School of Dentistry, University of Ribeirao Preto, Ribeirao Preto, Sao Paulo, Brazil; 2 MSc, Biotechnology Division, University of Ribeirao Preto, Ribeirao Preto, Sao Paulo, Brazil; 3 PhD, Biotechnology Division, University of Ribeirao Preto, Ribeirao Preto, Sao Paulo, Brazil; 4 PhD, Oral Pathology Section, Federal University of Pernambuco, Recife, Pernambuco, Brazil

## Abstract

Objectives: The aim of this study was to evaluate, by the semi-quantitative histological analysis, the anti-inflammatory activity of the ethanolic extracts of Serjania erecta e Zeyheria Montana, in experimental pulpits in rats. Study Design: In order to induce pulp inflammation, cavities were performed on the occlusal surface of the mandibular first molars of 45 male rats, without pulp exposure. The animals were distributed into 4 groups: GI, teeth without cavities; GII, single dose of saline solution via intraperitoneal (IP); GIII, single dose (IP) of 300mg/Kg of ethanolic extract of Zeyheria montana; GIV, single dose (IP) of 300mg/Kg of ethanolic extract of Serjania erecta. After 6, 12 and 24 hours, 5 animals of each group were killed by anesthetic overdose. The histological analyses of the pulp tissue were performed and the data analyzed by Dunn´s multiple test, at significance of 5%. Results: After 12 h, the GIII presented score statistically lower (p<0.05) than positive control group. After 24 h, GIII presented inflammatory index statistically lower than the positive control (p<0.01) and Serjania erecta (p<0.05) groups. Conclusion: The Zeyheria montana extract presented better anti-inflammatory activity than positive control group and Serjania erecta extract, which did not show anti-inflammatory effect in the analyzed periods.

** Key words:**Anti-inflammatory effect, experimental pulpitis, histological analysis, phytotherapy, rats.

## Introduction

The majority of odontogenic pains are of inflammatory pulpal or periapical origin, mainly caused by dental caries (1). When pulpitis is involved, it is necessary to interrupt the process responsible for the tissue lesion, and consequently relieve the painful symptomatology that is almost always the patient’s main complaint. Anti-inflammatory and analgesic medications are drugs used in pain relief and inflammation, diminishing the symptoms of the tissue disorders. Notably, they act inhibiting enzymes involved in the synthesis of the different inflammatory mediators (2).

Non steroidal anti-inflammatory drugs (NSAIDS) are generally the most used to control odontogenic pain of inflammatory origin. These are the cyclooxigenase inhibitors 1 (COX-1) and 2 (COX-2). Some studies have shown evidence that both selective and non-selective COX-2 inhibitor medications are effective for the reduction of the inflammatory reaction in experimental pulpitis in rats (1).

Although there is a biodiversity rich in vegetable species and various plants present the potential to produce compounds capable of causing various pharmacological alterations, a large number of plants have not yet been studied with the aim of establishing new drugs or phytotherapies (3,4). In order to gain a better understanding about the therapeutic activity of plants, it is necessary to know their metabolites, the chemical compounds formed, degraded, or simply transformed by chemical reactions in the vegetable cell.

Serjania erecta belongs to the Sapindaceae family, which is widely distributed in the tropical regions of the world, being typical of the Brazilian “cerrado” – dry regions with stunted vegetation. Hydroalcoholic extracts of Serjania erecta have revealed the presence of flavonoids, saponines, tannins, steroids and triterpenoids, which justify their popular indication for the treatment of inflammatory and ulcerative diseases (5). Previous studies have shown that some species of the genus Serjania, including Serjania erecta, as well as some of the compounds isolated from them, present anti-inflammatory, analgesic, antibacterial and antifungal action (3-6).

Zeyheria montana belongs to the Bignoniaceae family, genus: Zeyheria Mart, tribe: Tecomeae Endl., species: Zeyheria montana Mart., has the synonym of Zeyhera. It is a shrub commonly found in the Brazilian dry regions, specifically in the mid- and southwestern regions of the country (7). The roots of Zeyheria montana are used in popular medicine to treat tumors of the skin, while the leaves are used to combat inflammations in general. This species produces terpenes and flavonoids (7). Guenka et al. (8) observed important antinociceptive and anti-inflammatory activity of the ethanol extract of Zeyheria montana, administered intraperitoneally.

In spite of the potential therapeutic effect of various phytotherapies, particularly the anti-inflammatory action of Serjania erecta and Zeyheria montana, these medications are scarcely used in Dentistry. There are no previous studies evaluating the an-ti-inflammatory effect of phytotherapeutic medications on the pulp tissue. Therefore, it becomes relevant to evaluate the possible anti-inflammatory effect of phytotherapeutic medications on pulp tissue.

## Material and Methods

This study was approved by the Animal Research Ethics Committee of the University of Ribeirao Preto, Sao Paulo, Brazil and the ethical concepts for use of laboratory animals were observed in all phases of the experiment.

Preparation of the Serjania erecta and Zeyheria montana extracts

Serjania erecta leaves were collected in Araxá, Minas Gerais, Brazil, in November 2005. Dried and pulverized leaves (200 g) were macerated in 95% ethanol (1.0 L) for 24 h at 25 oC, and filtrate was concentrated, lyophilized, and resuspended in distilled water prior to use. Zeyheria montana leaves were collected in Franca, Sao Paulo, Brazil, in October 2005. A proportion of 1 Kg of dried and powdered leaves were used to 5 liters of solvent (95% ethyl alcohol) and the vegetable matter remained in maceration for 24h at 25oC before each extraction. The filtrate was concentrated, lyophilized and resuspended in distilled water.

Both, Serjania erecta and Zeyheria montana, were identified at the Botanic Institute at the State University of Campinas (Campinas, SP, Brazil) and a vouchers specimen (840 for Serjania erecta and 830 for Zeyheria montana) are kept in the Herbarium of University of Ribeirão Preto. Both extracts were stored in suitable receptacles in the Medicinal Plant Laboratory of the University of Ribeirão Preto.

To prepare the solutions of the extracts, 900 mg of each extract individually were added to safe-lock microtubes (Eppendorf AG, Hamburg, Germany), previously tared, and then 1.2 ml of physiological solution (NaCl 0.9%) was added. To obtain a homogeneous solution, the extracts were mixed with the physiological solution with the use of a computerized ultrasonic cleaner - USC 700 (Ultrasonic Cleaner UNIQUE, Indaiatuba, SP, Brazil) and a tube agitator MA-162 vortex type (Marconi Equipamentos para Laboratório LTDA, Piracicaba, SP, Brazil). Thus, the vegetable extracts were obtained in a saline solution at the concentration of 750 mg/mL of Zeyheria montana and Serjania erecta.

Induction of Inflammatory Reaction in Rat Pulp Tissue

For the study, 45 male rats (Rattus novergicus albinus, Wistar), weighing between 200 and 250 grams were used. They were kept in an acclimatized room and received a balanced diet and water ad libitum.

The animals were anesthetized with ketamine chloride (0.1 mg/kg) (SESP-Divisão Vetbrands Saúde Animal, Jacareí, SP, Brazil) associated with xylazine (0.05 mg/kg) (Bayer do Brasil, Belford Roxo, RJ, Brazil) administered intramuscularly. Previous to procedure, to confirm the adequate anesthesia, the animals did not respond to any stimulus. In order to induce a pulp inflammation, a cavity was prepared on the occlusal surface, medial portion, of the both mandibular first molars by an air turbine (Dabi-Atlante, Ribeirão Preto, SP, Brazil) with a ½ tungsten carbide bur (KG Sorensen, São Paulo, SP, Brazil), under constant cooling. The depth of the cavity formed corresponded to the active diameter of the bur, without pulp exposure.

After the cavities were made, the animals were randomly distributed into 3 experimental groups with 5 animals each, according to the ethanol extract used: Group II – single dose of saline solution via intraperitoneal (IP) (positive control); Group III – single dose (IP) of ethanolic extract of Zeyheria montana (300mg/Kg) and Group IV – single dose (IP) of ethanolic extract of Serjania erecta (300mg/Kg). In addition, the intact mandibular second and third molars (without cavities) of the animals from the Group II were used as negative control (Group I).

Sixty minutes after cavities procedures, the animals of Groups III and IV received 300 mg/Kg of ethanol extract of Zeyheria montana and Serjania erecta, respectively, via IP. Saline physiological solution that was used for preparing the solutions of the extracts was administered via IP in equal volume in the animals of Groups I and II. The concentrations of the extracts and the intraperitoneal administration were chose because of the good anti-inflammatory effects previously obtained by Guenka et al. (8).

In the periods of 6, 12 and 24 hours after the intraperitoneal injection of the studied extracts, 5 animals of each group were killed by anesthetic overdose, establishing final distribution of the animals according to the drug used and the period analyzed. The mandibles were removed, fixed in a 10% neutral-buffered formalin solution (Merck KGaA, Darmstadt, Germany) for 48 hours and decalcified with an aqueous solution of 10% trichloroacetic acid (Merck KGaA, Darmstadt, Germany) for 24 hours, washed under running water for 12 hours and afterwards sectioned in the region of the mandibular molars, in the mesiodistal direction. Afterwards they were processed for conventional histological examination; serial histological cuts (6 µm thick) were obtained and stained with hematoxylin-eosin. Moreover, the cuts were stained with the Brown and Brenn method in order to certify the absence of bacterial contamination.

Histological Analysis 

Double-blind histologic analysis was performed in each specimen; a total of 25 cuts were analyzed for each sample. For semi-quantitative histologic analysis, the criteria proposed by McClanaham, et al. (9) were adapted, as follows:

Score 1- normal pulp – intact layer of odontoblasts, absence of inflammatory infiltrate, hyperemia or edema (0% of the inflammatory microscope field).

Score 2- mild infiltrate of inflammatory cells, neutrophils, lymphocytes (1-33% of the inflammatory microscope field), absence of edema or hyperemia.

Score 3- moderate infiltrate of inflammatory cells, neutrophils, lymphocytes and/or presence of edema and/or hyperemia or necrosis (34-66% of the microscope field).

Score 4- severe inflammation with predominance of inflammatory cells, neutrophils, lymphocytes and/or hyperemia, hemorrhage, necrosis and possible dystrophic calcification (67-100% of the microscope field).

The analyzed area coincided with the region immediately below the cavity. In the specimens of the Group I (without cavity), the analyzed area corresponded to pulp horn.

Statistical Analysis 

For statistical analysis, Kruskal-Wallis and Dunn’s multiple tests were applied with the aid of the software GraphPad InStat (GraphPad Software Inc, San Diego, USA) to verify whether there was statistically significant difference between the scores according to the studied groups, with a 5% level of significance.

## Results

The original scores for all animals of groups II, III and IV are listed in  Table 1. All samples presented negative for the Brown and Brenn staining method.

**Table 1:**
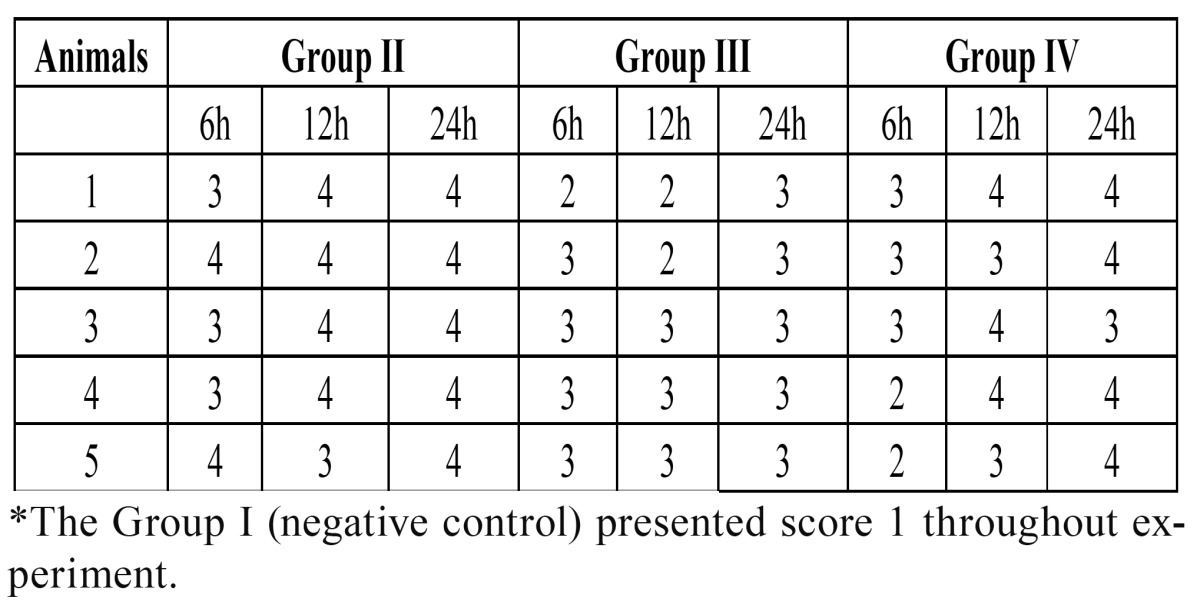
Original scores obtained after histological analysis in each animal from the Groups II (positive control), III (Zeyheria montana) and IV (Serjania erecta)*.

In Group I, which was composed of teeth that were not submitted to cavity preparation, in all periods, normal pulp tissue was observed, with an intact layer of odontoblasts, preserved architecture and absence of inflammatory infiltrate, hyperemia or edema.

**Figure 1:**
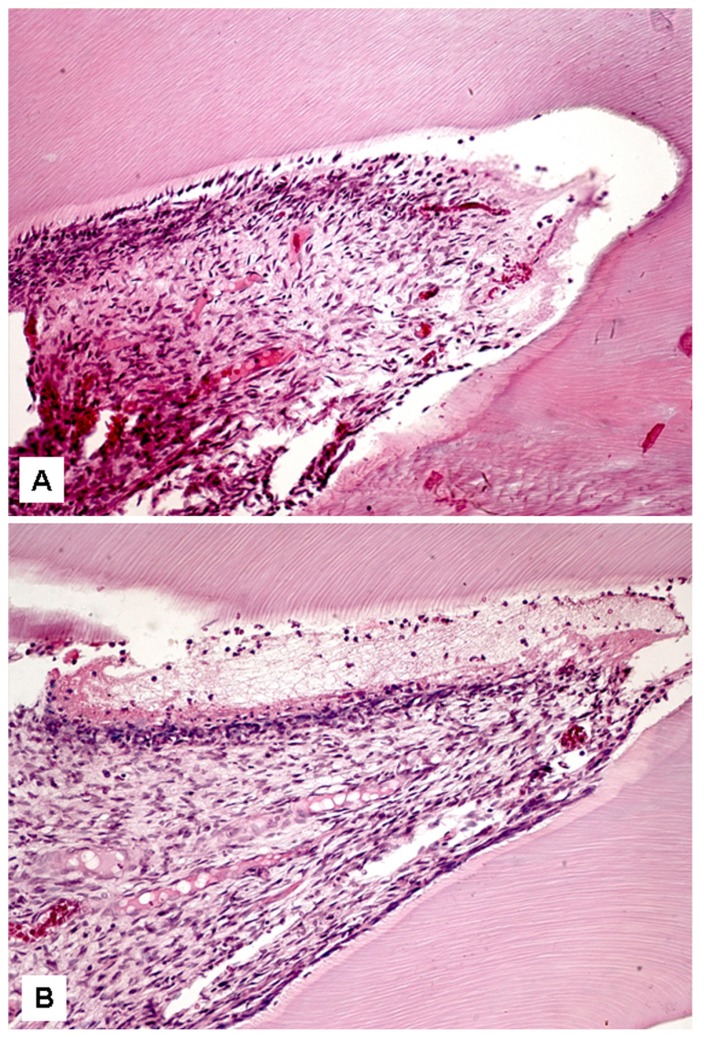
Pulp tissue after 6 hours. (A) Although there was a mild inflammation, discrete focus of necrosis and hyperemia were observed in a specimen of Zeyheria montana group (score 3). (B) Mild inflammation, edema and hyperemia were observed in this Serjania erecta specimen (score 3) (hematoxylin-eosin, original magnification, x200).

**Figure 2:**
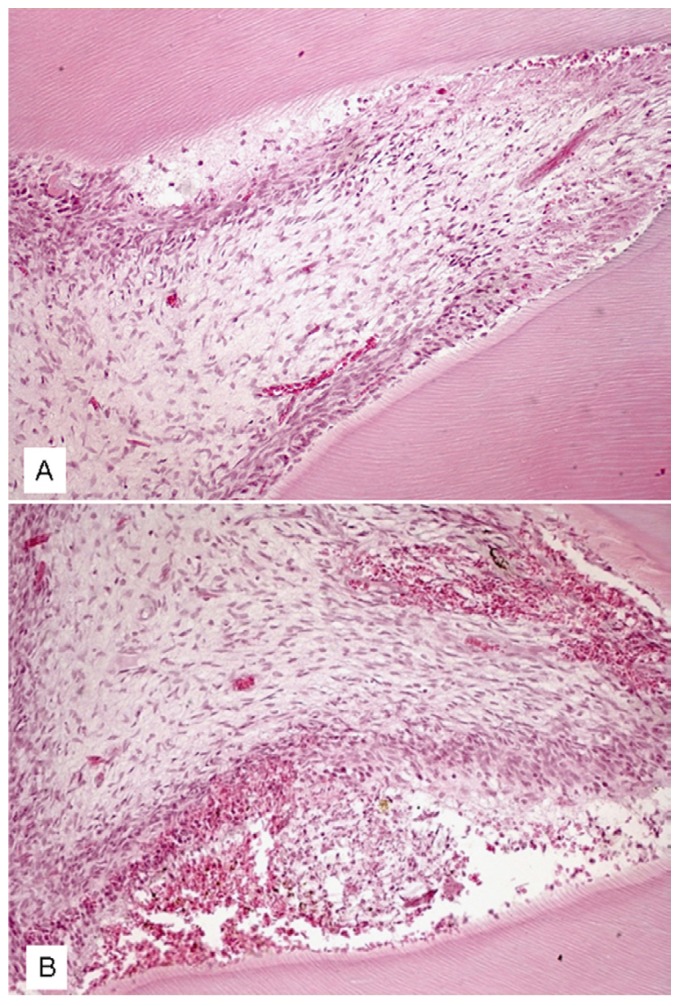
Pulp tissue after 12 hours. (A) Mild inflammation, edema and necrosis (up to 66% of the microscope field) in Zeyheria montana group (score 3). (B) Severe inflammation, with presence of edema and hemorrhage in Serjania erecta group (score 4) (hematoxylin-eosin, original magnification, x200).

**Figure 3:**
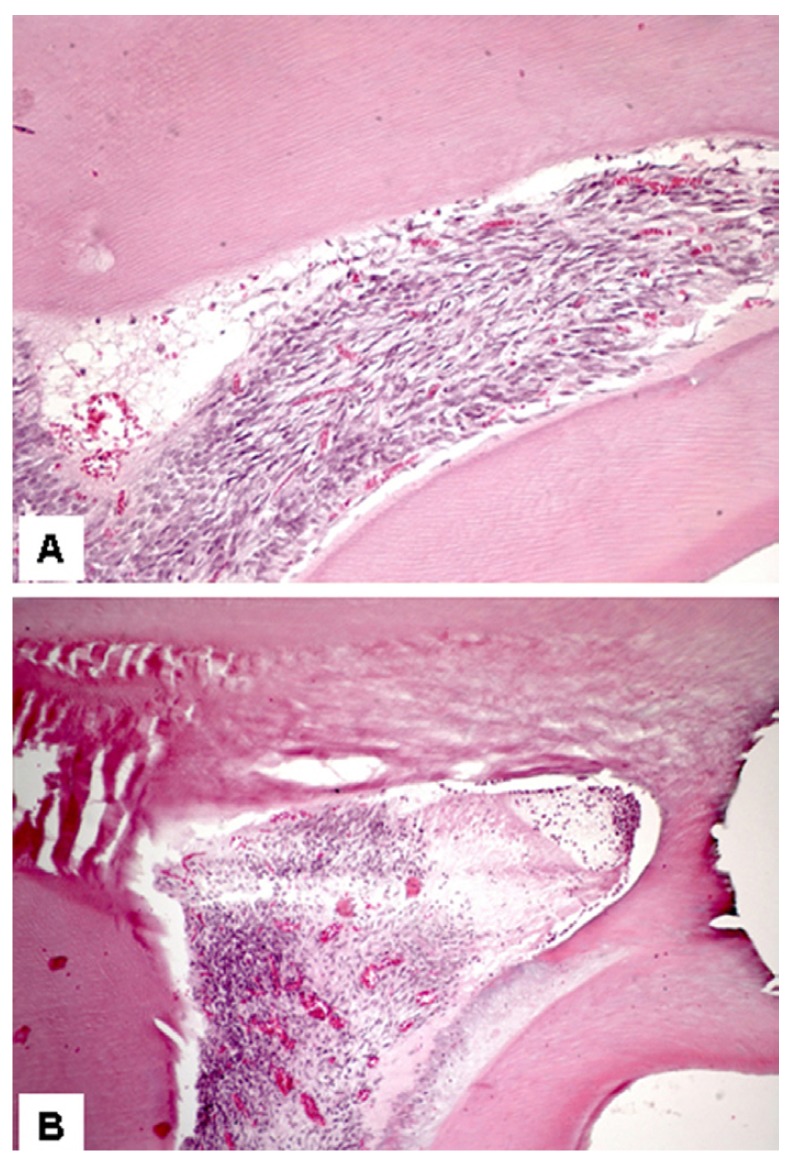
Pulp tissue after 24 hours. (A) Mild inflammation, edema, hyperemia were observed in Zeyheria montana group after 24 hours of treatment (score 3). No necrosis was observed (hematoxylin-eosin, original magnification, x200). (B) Severe inflammation, edema and necrosis were observed in Serjania erecta group after 24 hours of treatment (score 4) (hematoxylin-eosin, original magnification, x100).

After 6 hours, no statistically significant difference was observed among the groups, which presented similar results (Fig. 1). However, after 12 hours, the Zeyheria montana group (GIII) presented a significantly lower inflammatory index (p<0.05) than the positive control group (GII). However, the Zeyheria montana (GIII) (Fig. 2) and Serjania erecta (GIV) groups presented statistically similar indexes, although Serjania erect group had presented three specimens with severe inflammation (Fig. 2). After 24 hours, the Zeyheria montana group (Fig. 3) presented a significantly lower inflammatory index than the positive control (p<0.01) and Serjania erecta groups (p<0.05). The positive control and Serjania erecta groups presented statistically similar results. In all the periods, the negative control group presented a significantly lower score than all the other studied groups (p<0.05).

When the results obtained are compared by Dunn’s multiple comparisons test, irrespective of the periods studied, it was observed that the groups treated with Zeyheria montana presented significantly lower inflammatory indexes than the positive control group (p<0.05), while the Zeyheria montana and Serjania erecta groups presented statistically similar inflammatory indexes (p>0.05). The positive control and Serjania erecta groups presented statistically similar results (p>0.05). The negative control group presented significantly lower scores than the positive control (p<0.001), Zeyheria montana (p<0.01) and Serjania erecta groups (p<0.001).

## Discussion

To establish a safe and effective medication for the control of pulp inflammation and determine the effects of the analgesic and anti-inflammatory drugs available on the market, some studies have been conducted, using different study models, in humans and animals (1,10,11). Studies that evaluated the anti-inflammatory action of drugs on inflamed pulp tissues are scarce and the majority of them used NSAIDS available on the market, such as meloxicam, sodium diclofenac, ibuprofen, rofecoxib and celecoxib (1,12). Among these tested drugs, ibuprofen, rofecoxib and celecoxib presented good anti-inflammatory activity (1,12). Nevertheless, in the literature consulted, no studies evaluating the anti-inflammatory effect of phytotherapeutic medications on dental pulp were observed.

Comparing the anti-inflammatory activity of Zeyheria montana and Serjania erecta extracts in pulp, after 6 hours, the two groups presented statistically similar inflammatory indexes to those of the positive control. Twelve hours after the injection of the extracts, the Zeyheria montana group presented statistically lower inflammatory indexes than the positive control group, although it has presented statistically similar results to those of the Serjania erecta group. However, after 24 hours, the Zeyheria montana group presented a lower significantly inflammatory index than the control and Serjania erecta groups, with moderate chronic inflammatory reaction, edema, vascular congestions and absence of hemorrhage. Comparing the results of the groups irrespective of the periods, the Zeyheria montana group presented lower significantly inflammatory indexes than the positive control group, but similar results to those of the Serjania erecta group.

Zeyheria montana, belongs to the Bignoniaceae family and is an endemic medicinal species in the Brazilian dry regions. It presents various types of naphthoquinones, mainly lapaxol, in addition to other components such as flavonoids and terpenes (6). The animals treated with ethanol extract of Zeyheria montana presented lower pulp inflammatory indexes, when compared with the positive control and Serjania erecta groups. The presence of naphthoquinones and flavonoids in the Zeyheria montana extract (7) probably contributed to diminishing the pulp inflammatory reaction, particularly when compared with the control group, since these substances, especially the flavonoids, have the capacity to reduce the inflammatory reaction and inhibit lipid peroxidation, thus acting as antioxidant (13). Guenka et al. (8) observed that ethanol extract of Zeyheria montana presents antinociceptive and anti-inflammatory activities, which could be important for the pharmacological control of pain and inflammation.

The anti-inflammatory activity given by the flavonoids that are found in the studied plants, constituted mainly of rutin and catechin, is explained by their inhibitory effect on the metabolism of arachidonic acid (14,15). In fact, the anti-inflammatory activity given by some types of flavonoids is owing to the inhibition of prostaglandin E2 production (14,15).

Despite of the ethanol extract of Serjania erecta presents anti-inflammatory activity due to the presence of flavonoids, saponines, tannins, steroids and triterpenoids (4,5), in the present study the inflammatory indexes in the pulp tissue of the animals treated with Serjania erecta were statistically similar to those of the positive control group and different of the Zeyheria montana group. This finding probably is associated with the presence of several naphthoquinones in Zeyheria montana, mainly the lapachol, which presents significant anti-inflammatory activity (16). Moreover, if the time of analysis and the number of doses were increased, not only used for short periods of time as was done in the present study, perhaps the Serjania erecta extract would present a better performance.

Although the animals treated with Zeyheria montana extract presented lower pulp inflammation indexes than those treated with Serjania erecta, the present results did not show evidence of good anti-inflammatory effect of the extracts on pulp tissue, perhaps because of the characteristics of the pulp cavity itself, formed by hard and inelastic walls, in which the events that compose the inflammatory reaction, mainly edema, may cause great damage to the tissue, making it difficult for the studied extracts to act. In addition, the evaluation of inflammatory parameters in the pulp tissue is difficult. Although molecular techniques to assess the levels of cytokines and other inflammatory proteins are very useful, the histological analysis may provide valuable results.

In this study, the concentrations of the extracts and the intraperitoneal administration were chose because of the good an-ti-inflammatory effects obtained by Guenka et al. (8). However, for use in humans, extensive pharmacological and toxicological studies are necessary to test the safe, half-life and effectiveness of these phytotherapic agents, mainly when orally administered.

As there are no previous studies evaluating the anti-inflammatory effects of the plant extracts, particularly Serjania erecta and Zeyheria montana, in rat pulp tissues, it is difficult to make a comparison with other study models. In the methodology used, Zeyheria montana was shown to be more effective in the control of pulp inflammation than Serjania erecta. Undoubtedly, further studies must be conducted to evaluate other extracts, use other methodologies and thus test, understand and elucidate the effects of the various phytotherapeutical agents on pulp inflammatory reaction.
